# Hypertensive crisis occurs as a new aftermath beyond post-heatstroke: A case report and literature review

**DOI:** 10.1097/MD.0000000000043761

**Published:** 2025-08-01

**Authors:** Lei Li, Junda Cao, Fangqi Zhang

**Affiliations:** a Department of Thoracic Surgery, Wuhan Jinyintan Hospital, Tongji Medical College of Huazhong University of Science and Technology, Wuhan, China; b Hubei Clinical Research Center Diseases, Wuhan, China; c Wuhan Research for Infectious Center for Communicable Disease Diagnosis and Treatment, Chinese Academy of Medical Sciences, Wuhan, China; d Jiujiang City Key Laboratory of Cell Therapy, Department of Cardiovascular, The First Hospital of Jiujiang City, Jiujiang, China; e Department of Pulmonary and Critical Care Medicine, The 987th Hospital of Joint Logistics Support Force of People’s Liberation Army, Baoji, China.

**Keywords:** cardiovascular system, diagnosis, heatstroke, hypertensive

## Abstract

**Rationale:**

Heatstroke is a life-threatening condition and the most severe form of heat-related illness, characterized by a body temperature exceeding 40°C and multiorgan failure. In the cardiovascular system, hypovolemic shock is common. There is no report of hypertensive emergencies occurring during recovery from heatstroke.

**Patient concerns:**

This case study presents a 55-year-old male who was initially admitted with a lung abscess, which continued to improve during hospitalization, but he subsequently developed heatstroke due to outdoor activities in high-temperature environments. During recovery from multiple organ dysfunction caused by heatstroke, the patient experienced a month-long hypertensive emergency.

**Diagnoses:**

After ruling out hypertension caused by infection, the persistent hypertension in the patient was highly suspected to be caused by heatstroke.

**Interventions:**

The patient was transferred to an air-conditioned room, and symptomatic supportive treatment for heatstroke was initiated. Sodium nitroprusside was administered intravenously to treat the hypertensive crisis, while Nifedipine, Metoprolol, and Irbesartan were given orally for subsequent hypertension. Finally, the patient’s blood pressure gradually stabilized and remained so until the medications were discontinued.

**Outcomes:**

His symptoms basically disappeared within 1 month, and the patient did not report any discomfort with 8 months of follow-up.

**Lessons:**

This case was the first report of hypertensive emergencies during recovery from heatstroke. The possible mechanisms of hypertensive emergencies during recovery from heatstroke had been explored in this case study though literature review.

## 1. Introduction

Heat-induced diseases refer to a series of diseases caused by high temperature. As daily average temperatures rise, global heat-related deaths are expected to increase 4.7-fold by mid-century.^[1]^ Heatstroke is the severe form of heat-related illnesses, characterized by central nervous system dysfunction, multiorgan failure, and extreme hyperthermia (usually > 40°C).^[2,3]^ It is categorized as classic when it results from passive exposure to extreme environmental heat and as exertional when it develops during strenuous exercise.^[4]^ Classic heatstroke (CHS) occurs in epidemic form and contributes to 9% to 37% of heat-related fatalities during heatwaves, particularly among older individuals who often have preexisting illnesses. Exertional heatstroke sporadically affects predominantly young and healthy individuals, Including athletes, professional soldiers, outdoor workers, etc.

The primary pathogenic mechanism of heatstroke involves core body temperature continuing to rise, leading to a direct cytotoxic effect and an inflammatory response, creating a vicious cycle, and eventually causing multiorgan failure.^[5]^ In the cardiovascular system, hypovolemic shock and abnormal elevation of myocardial markers (CK, CK-MB, and cTnI) are common.^[6]^ Epidemiological studies showed that heat can result in ischemic heart disease, stroke, heart failure, hypovolemic shock, and arrhythmia.^[7]^ However, there was no report of hypertensive emergencies during recovery from heatstroke in the previous literatures.

## 2. Case presentation

**Figure 1.**
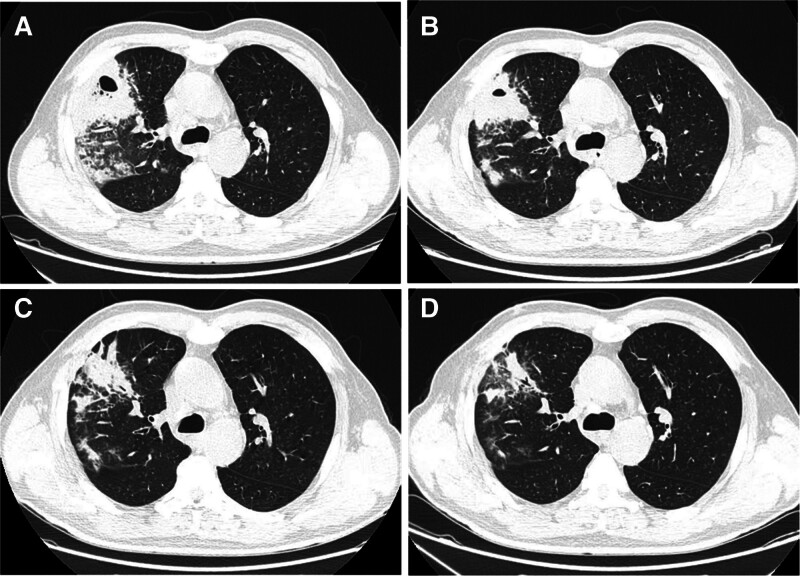
Patient’s computed tomography (CT) scan images at 4 time points. (A) Initial CT scan (D1) showed lung abscess in the right upper lung. (B) CT scan after antimicrobial treatment (D8) showed partial absorption of the lung abscess. (C) CT scan after re-onset of fever (D13) showed further absorption of the lung abscess. (D) Follow-up CT scan (D33) showed further improvement of the lung abscess.

A 55-year-old male was admitted to the hospital with cough and expectoration for 3 weeks, aggravated with fever for 1 week. The patient had a history of well-controlled type 2 diabetes for 6 months and smoking for 20 years. Other medical history such as hypertension was denied. He had no bird exposures, and no history of travel outside. At admission, the patient’s vital signs included body temperature of 38.7°C, respiratory rate of 25 times/min, pulse of 89 times/min, and blood pressure of 120/80 mm Hg. Physical examination showed no significant abnormalities. Laboratory tests and chest computed tomography (CT) is given in Table 1 and Figure 1A. The patient was diagnosed with lung abscess caused by *Klebsiella pneumoniae* (*K pneumonia*). And patient’s condition improved gradually by given antimicrobial treatment (Piperacillin/Tazobactam injection, 4.5 g, every 8 hours) and other comprehensive treatment measures, including airway clearance (Ambroxol, nebulized acetylcysteine solution, and postural expectoration) and glucose control (Insulin glargine and aspart). After 3 days of body temperature completely normal and respiratory symptoms were significantly relieved, that is, on the 8th day of admission, we reviewed the patient’s peripheral blood cells count, inflammation indicators, liver and kidney function and chest CT. Patient’s white blood cell, C-reactive protein, procalcitonin, liver and kidney function were completely normal (Table 2), chest CT also showed partial absorption of lung lesions (Fig. 1B).

**Table 1:** Laboratory parameters of patient at admission.

Laboratory parameters	At admission (d1–3)
WBC (NEU%)	14.52 × 10^9^/L (79.7%)
CRP	141.46 mg/L
PCT	0.21 ng/mL
ESR	110 mm/h
PPD	–
T-SPOT.TB	–
G/GM	–/–
(BALF) TB-DNA	–
(BALF) GM	–
Sputum culture	*Klebsiella pneumoniae*
(BALF) culture	*Klebsiella pneumoniae*
Blood culture	–
*Mycoplasma pneumoniae* IgM	–
*Chlamydia pneumoniae* IgM	–
CEA/NSE/SCC/CYFRA21-1	–/–/–/–
ANCA	All–
Antinuclear antibody spectrum	All–
AST/ALT	Normal range/normal range
Cr	Normal range
LDH	Normal range
CK-MB	Normal range
d-dimer	Normal range

ACNA = antineutrophil cytoplasmic antibodies, ALT = alanine aminotransferase, AST = aspartic transaminase, BALF = bronchoalveolar fluid, CEA/NSE/SCC/CYFRA21-1 = lung tumor markers, CK-MB = creatine kinase isoenzyme, Cr = creatinine, CRP = C-reactive protein, ESR = erythrocyte sedimentation rate, G = 1,3-beta-d-glucan, GM test = galactomannan test, LDH = lactate dehydrogenase, PCT = procalcitonin, PPD = purified protein derivative, TB-DNA = tubercle bacillus-DNA, T-SPOT.TB = T cell spots of tuberculosis infection, WBC = white blood cell.

**Table 2:** Changes in weather, clinical data, and main treatments of patient by time point.

	D1	D2	D3	D4	D5	D6	D7	D8	D9	D10	D11	D12	D13	D14	D15	D16	D17	D18	D19
Air temperature (°C)	33	28	25	25	26	29	31	33	35	37	34	26	29	34	34	30	33	37	35
Relative humidity (%)	67	82	97	100	99	100	83	88	87	85	86	95	82	69	68	70	92	69	76
Axillary temperature (°C)	38.7	37.9	37.1	36.4	36.7	36.2	36.1	36.5	40.9	40.2	38.7	38.3	37.5	36.8	36.4	36.2	36.7	36.8	36.6
Laboratory parameters																			
WBC (×10^9^/L) (NEU%)	14.52 (79.7%)							7.46 (67.2%)		2.82 (23.5%)		1.67 (26.3%)	9.69 (63%)	15.09 (76.1%)	14.46 (84.3%)		8.55 (64.6%)	7.68 (63.8%)	
PLT (×10^9^/L)	359							287		119		105	480	436	240		147	136	
CRP (mg/L)	141.46							6.01		90.93		57.97	30.56	16.49					
PCT (ng/mL)	0.21							0.15		0.58		4.74	0.58	0.19					
AST (U/L)	24							32		269		2093	1359	487	225		112	32	
ALT (U/L)	32							34		144		633	501	243	124		96	63	
Cr (µmol/L)	59							62		77		163	146	141	110		89	66	
LDH (U/L)	121							150				7673	6635	5258	3092		833	512	
CK (U/L)	72							86				1364	717	494	323		162		
CK-MB (U/L)	12							15				152	44	36	29		18		
d-dimer (mg/L)	0.26							0.12				>16	>16	>16	5.69		1.99	0.52	
Blood pressure (mm Hg)	124/74	130/70	122/72	126/74	128/72	130/82	134/84	130/90	140/90	132/88	120/72	140/88	138/84	220/120	160/100	160/100	160/100	140/90	140/90
Main treatments																			
	P/T	P/T	P/T	P/T	P/T	P/T	P/T	P/T	P/T	P/T	P/T	P/T	P/T	P/T	P/T	P/T	P/T	P/T	Lev
									APD	APD + PC	PC	ACR + PC + AFT	ACR + PC + AFT	Nit (8 µg/kg/min) + F(40 mg)	Nit (8 µg/kg/min) + F (40 mg)	Nit (6 µg/kg/min) + F (40 mg) + Nif (60 mg)	Nit (2 µg/kg/min) + F (20 mg) + Nif (60 mg) + Met (25 mg) + Irb (300 mg)	Nif (60 mg) + Met (25 mg) + Irb (300 mg)	Nif (60 mg) + Met (25 mg) + Irb (300 mg)
												FP + ATC	FP + ATC	FP + ATC	ATC	ATC	ATC	ATC	
												rhG-CSF							

ACR = air-conditioned room, AFT = aggressive fluid treatment, ALT = alanine aminotransferase, APD = antipyretic drugs, AST = aspartic transaminase, ATC = anticoagulation, CK-MB = creatine kinase isoenzyme, CRP = C-reactive protein, F = Furosemide, FP = fresh plasma, Irb = Irbesartan, LDH = lactate dehydrogenase, Lev = Levofloxacin, Met = Metoprolol, Nif = Nifedipine, Nit = Nitroprusside, P/T = Piperacillin/Tazobactam, PC = physical cooling, PCT = procalcitonin, PLT = platelet, rhG-CSF = recombinant human granulocyte colony-stimulating factor, WBC = white blood cell.

**Figure 2.**
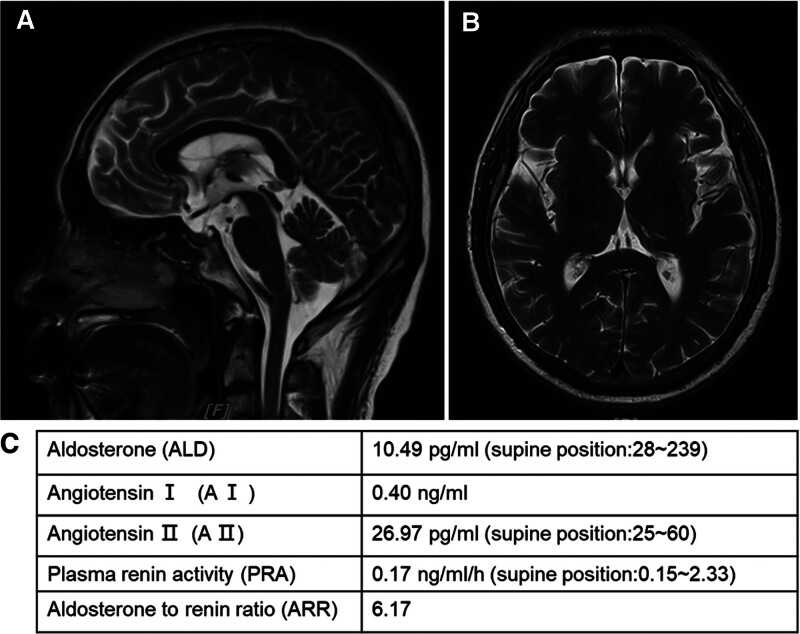
Patient’s brain MRI and hypertensive hormone testing after hypertensive emergencies. (A) Brain MRI showed Rathke’s cyst in the pituitary gland. (B) Brain MRI showed multiple lacunar infarctions in the thalamic region. (C) Renin–angiotensin system test showed no abnormalities.

However, on the 9th day of admission, the local temperature rose sharply by 12°C, and the patient engaged in some outdoor activities to deal with his personal affairs. Subsequently, the patient experienced fever again, with the highest temperature of 40.9°C, accompanied by dizziness, as well as fatigue, muscle soreness, nausea and vomiting after drinking pure water, but normal after drinking tea and beverages. In order to rule out acute upper respiratory tract infection, symptomatic antipyretic treatment was given and relevant examinations were improved. It is disappointing that nucleic acid tests for influenza A/B virus, Covid-19, adenovirus, respiratory syncytial virus, and rhinovirus were all negative. The next day (D10), the patient still continued to have high fever, and the antipyretic agents were not good, mainly based on physical cooling. Retests of peripheral blood cells count, C-reactive protein, procalcitonin, and liver function began to show abnormalities. On the day 11, the patient still had high fever and relied on physical cooling to avoid persistent high temperatures. Blood cultures and sputum cultures were negative. Plasmodium and Brucella was tested also, and these were also negative. On the day 12, the patient still had fever, the peak was lower than before. But patient’s peripheral blood cells count, inflammatory indicators and organ functions further deteriorated. Through multidisciplinary team consultations, the patient’s fever on the one hand considered the manifestation of sepsis caused by uncontrolled lung abscess. It is recommended to perform next-generation sequencing (NGS) testing in blood and bronchoalveolar fluid to identify possible pathogenic bacteria, and review chest CT. On the other hand, based on the heat waves, CHS could not be ruled out. The patient was transferred to an air-conditioned room and symptomatic supportive treatment such as aggressive fluid treatment and diuresis, infusion of fresh plasma, anticoagulation and anti-inflammation were actively taken. On day 13, no pathogens were detected in the patient’s blood metagenomic NGS and no other pathogens were detected in the bronchoalveolar fluid tNGS except for *K pneumonia*. And reexamination chest CT showed further absorption of the lung abscess (Fig. 1C). On the day 14, however, the patient developed hypertensive emergencies (BP 220/120 mm Hg, no significant difference in the major arteries of the limbs) without obvious other concomitant symptoms. Sodium nitroprusside was given intravenously to reduce blood pressure and the cause of hypertensive emergencies was actively sought. Unfortunately, no significant abnormalities were found on cardiac ultrasound, renal ultrasound, renal artery ultrasound, adrenal ultrasound and deep vein ultrasound. Brain MRI and hypertensive hormone (renin, angiotensin, and aldosterone) testing also cannot explain the hypertensive emergencies (Fig. 2).

Then, heatstroke was diagnosed by multidisciplinary team consultations. By active treatment of heatstroke, the patient’s fever and accompanying symptoms were quickly relieved, and abnormal organ function indicators also gradually decreased. However, the patient still had to relied on sodium nitroprusside to maintain relatively non-dangerous blood pressure for the following 2 days. And began to combine other antihypertension drugs to reduce blood pressure synergistically. On day 17, sodium nitroprusside was stopped and oral drugs were used to reduce blood pressure. On day 19, the patient was discharged from the hospital and continued to take oral drugs to reduce blood pressure. Two weeks after discharge, the patient gradually reduced antihypertension medications until they stopped and blood pressure remained stable. Follow-up chest CT (Fig. 1D) showed that the patient’s lung abscess was further absorbed, peripheral blood cells count, inflammation indicators, liver and kidney functions, and coagulation function were normal, and blood pressure was stable. Changes in weather, clinical data, and main treatments are shown in Table 2. The patient did not report any discomfort with 8 months of follow-up. The patient expressed satisfaction with the doctor’s diagnosis and treatment.

## 3. Discussion

Heatstroke is a life-threatening and the most severe form of heat-related illnesses, characterized by central nervous system dysfunction, multiorgan failure, and extreme hyperthermia (usually > 40°C).^[8]^ Depending on its cause, heatstroke may be categorized as either classic or exertional. CHS frequently occurs as an epidemic among elderly persons whose ability to adjust physiologically to heat stress has become compromised, chronically ill persons, and those who cannot care for themselves.^[9]^ Multiple intrinsic physiological, social, and medical risk factors render elderly persons more vulnerable to ongoing heat owing to their diminished thermoregulatory capacity.^[9,10]^

In this case, the patient was hospitalized due to a lung abscess and had underlying diabetes. The patient’s lung abscess continued to improve after anti-infective treatment, the patient’s blood pressure remained normal throughout during this process. After engaged in some outdoor activities in a local temperature rose sharply day, the patient was fever again, accompanied by dizziness, as well as fatigue, muscle soreness, nausea and vomiting. Strengthening anti-infection treatment is ineffective in alleviating these symptoms. Laboratory tests also failed to reveal any signs of aggravation of the infection indicators. After transfer to an air-conditioned room, and symptomatic supportive treatment for heatstroke, the patient’s fever and accompanying symptoms were quickly relieved. But he occurs hypertensive crisis. Subsequently, antihypertensive drugs to treat the hypertensive crisis. Finally, the patient’s blood pressure gradually stabilized and remained so until the medications were discontinued.

Classic heatstroke can be diagnosed for this patient. The primary pathogenic mechanism of heat stroke involves 2 aspects, the direct cytotoxic effect caused by the increase of core temperature, and heatstroke-related inflammatory response (heat-sepsis).^[5,6,11]^ Circulatory disorders in patients with heat stroke are mainly manifested as blood volume deficiency and cardiac dysfunction.^[12–14]^ There is no report of hypertensive emergencies in recovery period of multiple organ dysfunction caused by heatstroke. In patients presenting with malignant hypertension, secondary causes can be found in 20% to 40% and most often consist of renal parenchymal disease and renal artery stenosis.^[15]^ Marked activation of the renin–angiotensin system is often present.^[16]^ In this case, hypertensive emergencies suddenly occurred when multiple organ functions gradually recovered after heat stroke. Relevant hypertension examinations like renal ultrasound, renal artery ultrasound, and renin–angiotensin system were completed and no obvious causes were found. In addition, the patient’s blood pressure gradually returned to normal within 1 month.

The occurrence of persistent hypertension after heat stroke cannot be explained, but it can be distinguished from those with transient hypertension. Transient hypertension emergencies can occur during the recovery phase from heatstroke, a condition characterized by a significant elevation in body temperature due to prolonged exposure to high environmental temperatures or intense physical exertion. The physiological stress imposed by heatstroke can lead to various cardiovascular complications, including hypertensive crises. One of the primary causes of hypertensive emergencies in the context of heatstroke is the dysregulation of the body’s thermoregulatory and cardiovascular systems. During heat exposure, the body attempts to dissipate heat through vasodilation and increased heart rate. However, this compensatory mechanism can become overwhelmed, particularly in individuals with preexisting cardiovascular conditions or those who are dehydrated. The resultant stress can lead to endothelial dysfunction and increased vascular resistance, contributing to acute hypertension during recovery phases.^[17,18]^

Additionally, the inflammatory response triggered by heatstroke can exacerbate hypertensive episodes. Studies have shown that hypertensive emergencies are associated with elevated markers of inflammation and coagulation, which can further compromise vascular integrity and function. For instance, increased levels of C-reactive protein and other inflammatory markers have been documented in patients experiencing hypertensive crises, suggesting a link between systemic inflammation and acute hypertension.^[19]^ This inflammatory state may be particularly pronounced following heatstroke, where the body is already under significant physiological stress.

Moreover, the role of fluid and electrolyte imbalances cannot be overlooked. Heatstroke often leads to dehydration and electrolyte disturbances, which can precipitate hypertensive emergencies. The loss of fluids can result in reduced blood volume, prompting compensatory mechanisms that may ultimately lead to increased blood pressure as the body attempts to restore homeostasis. In this context, the management of hydration and electrolyte levels becomes critical in preventing hypertensive crises during recovery from heat-related illnesses.^[20,21]^

It is worth noting that the diagnosis of specific types of hypertensions is also of great significance. Speckle tracking echocardiography (STE) has emerged as a valuable tool in enhancing the prognostic risk stratification of patients with acute cardiovascular diseases. This advanced imaging technique allows for the detailed assessment of myocardial deformation, providing insights that are often not captured by traditional echocardiographic methods. The ability of STE to evaluate myocardial strain and strain rate offers clinicians a more sensitive measure of cardiac function, which is crucial in the context of acute cardiovascular events. Some studies highlight the utility of STE in the context of acute myocardial infarction and chronic coronary artery disease.^[22]^ This suggests that STE can provide additional prognostic information beyond traditional measures such as ejection fraction, particularly in chronic conditions where myocardial deformation may be subtly impaired. The integration of speckle tracking echocardiography into clinical practice offers a significant advancement in the prognostic assessment of patients with acute cardiovascular diseases.^[23]^ By providing a more detailed evaluation of myocardial function, STE enhances the ability to identify high-risk patients and tailor management strategies accordingly.

## 4. Conclusion

The causes of hypertensive emergencies during recovery from heatstroke are multifactorial, involving dysregulation of cardiovascular responses, inflammatory processes, and fluid imbalances. However, these hypertensions that occurred after heat stroke were transient and transient in the previous literature. High blood pressure after heatstroke lasting as long as 1 month has not been reported. A comprehensive understanding of these mechanisms is essential for healthcare providers to effectively monitor and manage patients recovering from heatstroke, ensuring timely interventions to mitigate the risks of hypertensive crises.

## Author contributions

**Conceptualization:** Lei Li.

**Data curation:** Junda Cao, Fangqi Zhang.

**Formal analysis:** Lei Li.

**Investigation:** Lei Li.

**Methodology:** Lei Li, Junda Cao, Fangqi Zhang.

**Project administration:** Fangqi Zhang.

**Supervision:** Lei Li.

**Writing – review & editing:** Lei Li.

**Writing – original draft:** Junda Cao, Fangqi Zhang.

## References

[1] Romanello M Napoli CD Green C. The 2023 report of the Lancet Countdown on health and climate change: the imperative for a health-centred response in a world facing irreversible harms. Lancet. 2023;402:2346–94. 37977174 10.1016/S0140-6736(23)01859-7 PMC7616810

[2] Roberts WO Armstrong LE Sawka MN Yeargin SW Heled Y O’Connor FG. ACSM expert consensus statement on exertional heat illness: recognition, management, and return to activity. Curr Sports Med Rep. 2023;22:134–49. Published April 1, 2023. 37036463 10.1249/JSR.0000000000001058

[3] Bauman J Spano S Storkan M. Heat-related illnesses. Emerg Med Clin North Am. 2024;42:485–92. 38925769 10.1016/j.emc.2024.02.010

[4] Bouchama A Abuyassin B Lehe C. Classic and exertional heatstroke. Nat Rev Dis Primers. 2022;8:8. 35115565 10.1038/s41572-021-00334-6

[5] Epstein Y Yanovich R. Heatstroke. N Engl J Med. 2019;380:2449–59. 31216400 10.1056/NEJMra1810762

[6] Epstein Y Roberts WO. The pathophysiology of heat stroke: an integrative view of the final common pathway. Scand J Med Sci Sports. 2011;21:742–8. 21635561 10.1111/j.1600-0838.2011.01333.x

[7] Desai Y Khraishah H Alahmad B. Heat and the heart. Yale J Biol Med. 2023;96:197–203. Published June 30, 2023. 37396980 10.59249/HGAL4894 PMC10303253

[8] Leon LR Bouchama A. Heat stroke. Compr Physiol. 2015;5:611–47. 25880507 10.1002/cphy.c140017

[9] Kravchenko J Abernethy AP Fawzy M Lyerly HK. Minimization of heatwave morbidity and mortality. Am J Prev Med. 2013;44:274–82. 23415125 10.1016/j.amepre.2012.11.015

[10] Kenney WL Craighead DH Alexander LM. Heat waves, aging, and human cardiovascular health. Med Sci Sports Exerc. 2014;46:1891–9. 24598696 10.1249/MSS.0000000000000325 PMC4155032

[11] Lim CL. Heat sepsis precedes heat toxicity in the pathophysiology of heat stroke-a new paradigm on an ancient disease. Antioxidants (Basel). 2018;7:149. 30366410 10.3390/antiox7110149 PMC6262330

[12] Quinn CM Audet GN Charkoudian N Leon LR. Cardiovascular and thermoregulatory dysregulation over 24 h following acute heat stress in rats. Am J Physiol Heart Circ Physiol. 2015;309:H557–64. 26071550 10.1152/ajpheart.00918.2014

[13] Wang X Yuan B Dong W. Humid heat exposure induced oxidative stress and apoptosis in cardiomyocytes through the angiotensin II signaling pathway. Heart Vessels. 2015;30:396–405. 24898407 10.1007/s00380-014-0523-6

[14] Crandall CG Wilson TE. Human cardiovascular responses to passive heat stress. Compr Physiol. 2015;5:17–43. 25589263 10.1002/cphy.c140015 PMC4950975

[15] van den Born BH Lip GYH Brguljan-Hitij J. ESC Council on hypertension position document on the management of hypertensive emergencies [published correction appears in Eur Heart J Cardiovasc Pharmacother. 2019, 5(1):46. doi: 10.1093/ehjcvp/pvy040]. Eur Heart J Cardiovasc Pharmacother. 2019;5:37–46. 30165588 10.1093/ehjcvp/pvy032 PMC13588668

[16] van den Born BJ Koopmans RP van Montfrans GA. The renin–angiotensin system in malignant hypertension revisited: plasma renin activity, microangiopathic hemolysis, and renal failure in malignant hypertension. Am J Hypertens. 2007;20:900–6. 17679041 10.1016/j.amjhyper.2007.02.018

[17] Zhong L Ji J Wang C Liu Z. Clinical characteristics and risk factors of male exertional heatstroke in patients with myocardial injury: an over 10-year retrospective cohort study. Int J Hyperthermia. 2021;38:970–5. 34157921 10.1080/02656736.2021.1941312

[18] Casa DJ Armstrong LE Kenny GP O’Connor FG Huggins RA. Exertional heat stroke: new concepts regarding cause and care. Curr Sports Med Rep. 2012;11:115–23. 22580488 10.1249/JSR.0b013e31825615cc

[19] Derhaschnig U Testori C Riedmueller E Aschauer S Wolzt M Jilma B. Hypertensive emergencies are associated with elevated markers of inflammation, coagulation, platelet activation and fibrinolysis. J Hum Hypertens. 2013;27:368–73. 23254594 10.1038/jhh.2012.53

[20] Kotruchin P Pratoomrat W Mitsungnern T Khamsai S Imoun S. Clinical treatment outcomes of hypertensive emergency patients: results from the hypertension registry program in Northeastern Thailand. J Clin Hypertens (Greenwich). 2021;23:621–7. 33615688 10.1111/jch.14119 PMC8029559

[21] Li X Xv F Ma LZ. Acquired heat acclimation in rats subjected to physical exercise under environmental heat stress alleviates brain injury caused by exertional heat stroke. Brain Res. 2023;1811:148393. 37150340 10.1016/j.brainres.2023.148393

[22] Scharrenbroich J Hamada S Keszei A. Use of two-dimensional speckle tracking echocardiography to predict cardiac events: comparison of patients with acute myocardial infarction and chronic coronary artery disease. Clin Cardiol. 2018;41:111–8. 29359809 10.1002/clc.22860 PMC6489831

[23] Sonaglioni A Cara MD Nicolosi GL. Rapid risk stratification of acute ischemic stroke patients in the emergency department: the incremental prognostic role of left atrial reservoir strain. J Stroke Cerebrovasc Dis. 2021;30:106100. 34525440 10.1016/j.jstrokecerebrovasdis.2021.106100

